# Caution in Interpreting Results from Imputation Analysis When Linkage Disequilibrium Extends over a Large Distance: A Case Study on Venous Thrombosis

**DOI:** 10.1371/journal.pone.0038538

**Published:** 2012-06-04

**Authors:** Marine Germain, Noémie Saut, Tiphaine Oudot-Mellakh, Luc Letenneur, Anne-Marie Dupuy, Marion Bertrand, Marie-Christine Alessi, Jean-Charles Lambert, Diana Zelenika, Joseph Emmerich, Laurence Tiret, Francois Cambien, Mark Lathrop, Philippe Amouyel, Pierre-Emmanuel Morange, David-Alexandre Trégouët

**Affiliations:** 1 INSERM UMR_S 937, ICAN Institute, Université Pierre et Marie Curie, Paris, France; 2 INSERM UMR_S 1062, Université de la Méditerranée, Marseille France; 3 INSERM UMR_S 897, Université Victor Segalen, Bordeaux, France; 4 INSERM UMR_S 888, Hôpital La Colombière, Montpellier, France; 5 INSERM UMR_S 708, Université Pierre et Marie Curie, Paris, France; 6 INSERM UMR_S 744, Institut Pasteur de Lille, Université de Lille Nord de France, Lille, France; 7 Commissariat à l'Energie Atomique, Institut de Génomique, Centre National de Génotypage, Evry, France; 8 INSERM UMR_S 765, Hôpital Européen Georges-Pompidou, Université Paris-Descartes, Paris, France; 9 Centre Hospitalier Régional Universitaire de Lille, Lille, France; University of North Carolina, United States of America

## Abstract

By applying an imputation strategy based on the 1000 Genomes project to two genome-wide association studies (GWAS), we detected a susceptibility locus for venous thrombosis on chromosome 11p11.2 that was missed by previous GWAS analyses that had been conducted on the same datasets. A comprehensive linkage disequilibrium and haplotype analysis of the whole locus where twelve SNPs exhibited association p-values lower than 2.23 10^−11^ and the use of independent case-control samples demonstrated that the culprit variant was a rare variant located ∼1 Mb away from the original hits, not tagged by current genome-wide genotyping arrays and even not well imputed in the original GWAS samples. This variant was in fact the rs1799963, also known as the FII G20210A prothrombin mutation. This work may be of major interest not only for its scientific impact but also for its methodological findings.

## Introduction

We had previously reported the results of two genome-wide association studies (GWAS) for venous thrombosis (VT) conducted in samples of French origin [1], [2]. The first GWAS included 419 VT patients and 1,228 controls genotyped with the Illumina Sentrix HumanHap 300 beadchip [1] while the second, composed of 1,542 cases and 1,110 controls were genotyped with Illumina Human 610Quad and 660W beadchips [2]. In both studies, cases were unrelated VT patients, free of any chronic conditions and without known major genetic risk factors including anti-thrombin, protein C or protein S deficiency, homozygosity for FV Leiden and FII G2021A mutations. Controls were healthy individuals selected from two French national cohorts, the SUVIMAX [3] and Three-City Study (3C) [4], respectively. The meta-analysis of these two GWAS identified the well-established *ABO*, *F5*, *F11* and *FGG* genes and provided novel strong support in favor of *HIVEP1*, *PROCR* and *STAB2* loci as VT susceptibility genes [2]. This meta-analysis was performed using imputed data derived from the HapMap2 release 21 reference dataset containing 2,557,252 autosomal single nucleotide polymorphisms (SNPs). This genotype resource, used in a very large number of meta-GWAS analyses, is particularly efficient for testing the association with a phenotype of non genotyped SNPs whose minor allele frequencies (MAF) are greater than 5% [5]. However, it may not be adapted to infer the association of SNPs with lower MAF. To overcome this limitation and make possible the inference of rare SNPs with MAF as low as 1%, imputation analysis using data from the 1000 Genomes project [6] has recently been proposed, and a few examples [7]–[10] substantiated the interest of such approach.

**Figure 1:**
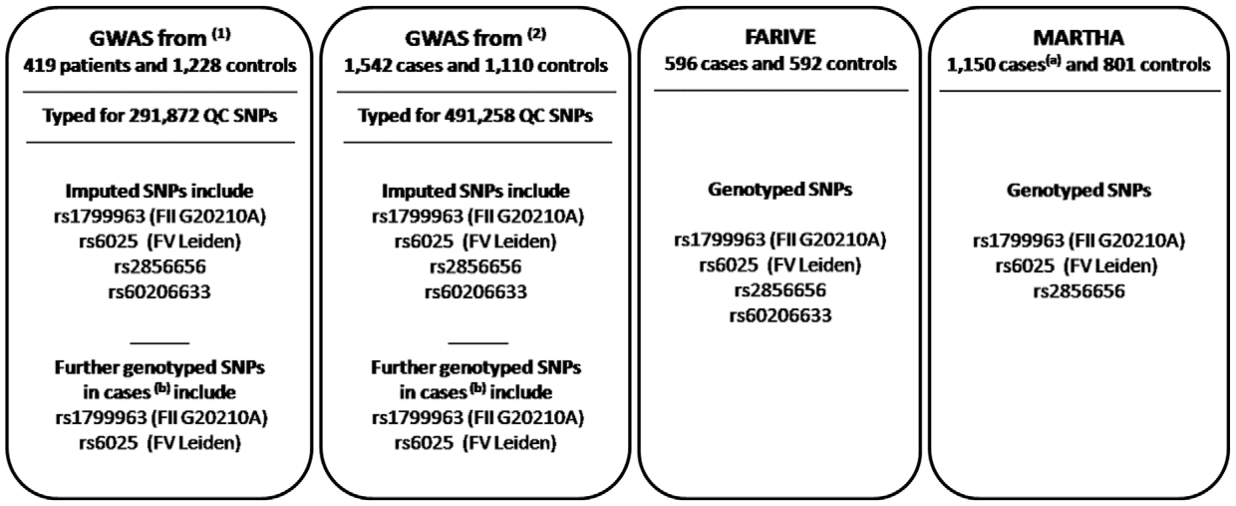
Case-control samples available in this work. ^(1)^ These individuals were typed with the Illumina Sentrix HumanHap300 beadchip containing 317,319 SNPs among which 291,872 satisfied the quality control (QC) criteria (Trégouët et al. (2009) Blood 113: 5298–5303). ^(2)^ These individuals were typed with the Illumina 610-Quad and Illumina 660W-Quad beadchips. Among the 551,141 SNPs common to both assays, 491,258 SNPs satisfied the QC criteria (Germain et al (2011) Plos One 6: e25581). ^(a)^ 812 VT patients of the MARTHA study were part of the GWAS^(2)^ VT sample. ^(b)^ The FV Leiden and FII G20210A mutations were genotyped in the GWAS patients as part of the study design where patients homozygous for these mutations were excluded.

Therefore, in this work, we re-analyzed these two GWAS on VT based now on the 1000G 2010-08 release containing 11,572,501 autosomal SNPs, and validated novel results in two additional case-control samples. A brief summary of the available samples used in this work is shown in Figure 1.

## Results

**Figure 2:**
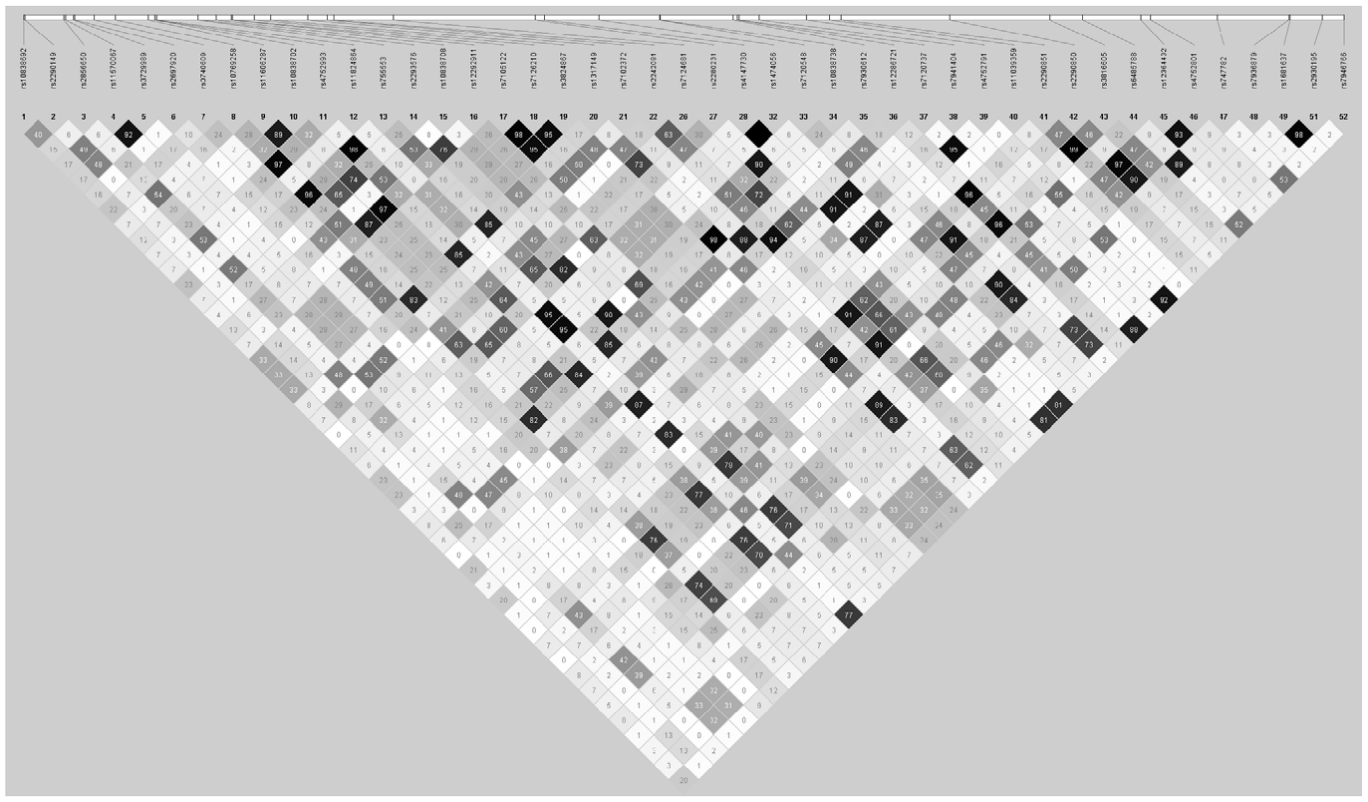
Pairwise linkage disequilibrium r^2^ between genotyped SNPs at the 11p11.2 locus over the 47,373,425–48,064,194 bp region in the second GWAS study (Germain et al. Plos One 2011).

The imputation analysis was performed using MACH [11] (v1.0.16a) (http://www.sph.umich.edu/csg/abecasis/mach/) and Minimac (v4.4.3) (http://genome.sph.umich.edu/wki/Minimac) software. 6,754,935 SNPs were imputed with good imputation quality (r^2^>0.3) [11] in both GWAS. The allele frequency distribution of the imputed studied SNPs was shown in Figure S1. Association of imputed SNPs with VT was tested using a likelihood ratio test statistics implemented in the mach2dat (v1.0.18) software (http://www.sph.umich.edu/csg/abecasis/mach/) while adjusting for the first four principal components as described in [2], separately in each GWAS. The results obtained in the two GWAS were then combined using a fixed-effect meta-analysis based methodology implemented in the METAL software [12]. A Quantile-Quantile (QQ) plot representation of the results was shown in Figure S2 and the resulting genomic control factor was 0.993. 217 SNPs were found, at the genome-wide significant at the 7.4×10^−9^ level, consistently associated with VT across the two GWAS samples (Table S1). These VT-associated SNPs overlapped five loci on four chromosomes. Four of the loci mapped the aforementioned *ABO*, *F5*, *F11* and *FGG* genes while a novel association involving the 11p11.2 locus (Table 1) was identified. Twelve SNPs, from position 47,373,425 to 48,064,194 (according to hg19 reference) and overlapping the *MYBPC3*-*SPI1*-*CELF1*-*KBTBD4*-*NUP160*-*PTPRJ* gene cluster demonstrated significant associations with VT, ranging from P = 6.97 10^−13^ to P = 2.23 10^−11^. All these SNPs had similar allele frequencies (∼3%) and similar genetic effects on VT risk (Odds Ratio (OR) ∼ 2.5) suggesting the existence of a strong linkage disequilibrium (LD) block (pairwise r^2^ or |D'|>0.8) explaining the association signal observed at the 11p11.2 locus. This hypothesis was supported by the results of a series of conditional logistic analyses showing that after adjusting on any of these SNPs all other observed associations at this locus vanished (P>0.10). Conversely, genotyped SNPs at this locus exhibited low to moderate LD (Figure 2) with median and 90^th^ percentile of the pairwise r^2^ distribution being 0.10 and 0.52, respectively. We further investigated the haplotype structure derived from the genotyped SNPs using a previously described statistical methodology [13] based on the Stochastic-EM algorithm [14]. For this, an Akaike Information Criterion (AIC) based strategy was applied to our largest GWAS [2] in order to identify the most informative and parsimonious haplotypic model of 1 to 4 genotyped SNPs, mapping 47,300,000 to 48,100,000, with respect to disease risk prediction. The best identified combination included three SNPs, rs2856650, rs3740689 and rs10769258, that generated six haplotypes whose global distribution strongly differed between cases and controls, consistently in both GWAS (Table 2). The haplotypic association appeared to be mainly attributable to the uncommon AGT haplotype that was more frequent in cases than in controls. The Odds Ratio for VT associated with this AGT haplotype was 2.37 [1.36–4.15] (P = 2.39 10^−3^) and 2.99 [2.02–4.44] (P = 4.23 10^−8^) in the first and second GWAS, respectively) (Table 2). When haplotype analyses were adjusted for the imputed dose at any of the twelve SNPs, these ORs were reduced to 1.03 [0.41–2.58] (P = 0.950) and 0.96 [0.55–1.66] (P = 0.879) suggesting that the AGT haplotype actually tagged the rare alleles of the long range LD block.

**Table 1:** Genome-wide significant association (p<1.×10^−8^) observed at the 11p11.2 locus.

				GWAS from ^2^					GWAS from ^1^					Combined
Locus	Position	SNP^(a)^		MAF^(b)^	logOR	se (LogOR)	P^(c)^	r^2 (d)^	MAF	logOR	se (LogOR)	P	r^2^	P^(e)^
MYBPC3	47373425	rs2856656	A/G	0.032	1.598	0.257	2.76 10^–12^	.8237	0.016	1.242	0.347	4.82 10^−4^	.7624	1.03 10^−12^
SPI1	47389771	chr11:47389771	C/G	0.035	1.628	0.247	1.09 10^−13^	.8371	0.018	1.044	0.328	1.92 10^−3^	.7719	6.97 10^−13^
CELF1	47565138	chr11:47565138	T/C	0.031	1.748	0.27	5.13 10^−13^	.7389	0.015	1.075	0.367	4.21 10^−3^	.7162	3.64 10^−12^
	47574081	rs60206633	C/G	0.031	1.73	0.267	5.47 10^−13^	.7444	0.016	1.061	0.364	4.41 10^−3^	.7211	3.69 10^−12^
KBTBD4	47598481	chr11:47598481	G/A	0.031	1.735	0.268	4.30 10^−13^	.7584	0.016	0.964	0.368	1.06 10^−2^	.7286	1.24 10^−11^
FNBP4	47764906	rs78261087	G/A	0.034	1.539	0.24	1.08 10^−12^	.8358	0.017	0.864	0.339	1.29 10^−2^	.7912	1.99 10^−11^
	47783614	chr11:47783614	T/C	0.034	1.528	0.239	1.11 10^−12^	.8417	0.017	0.858	0.336	1.30 10^−2^	.7957	2.23 10^−11^
NUP160	47818310	rs58883118	C/T	0.035	1.526	0.237	8.22 10^−13^	.8520	0.017	0.86	0.332	1.18 10^−2^	.8166	1.52 10^−11^
	47920993	chr11:47920993	G/T	0.034	1.411	0.232	2.06 10^−11^	.8796	0.016	1.048	0.333	2.19 10^−3^	.8525	1.13 10^−11^
	47947894	chr11:47947894	C/T	0.022	2.153	0.351	4.27 10^−12^	.6464	0.011	1.45	0.465	2.42 10^−3^	.6459	1.25 10^−11^
PTPRJ	48052066	rs117784795	C/T	0.039	1.315	0.207	2.67 10^−12^	.9417	0.020	0.775	0.29	9.28 10^−3^	.8986	1.78 10^−11^
	48064194	chr11:48064194	G/A	0.038	1.311	0.207	2.94 10^−12^	.9746	0.020	0.856	0.296	4.99 10^−3^	.9286	7.52 10^−12^

(a) SNPs for which no rsID has yet been allocated are named according to their position on chromosome 11.

(b) Minor Allele Frequency.

(c) P-value of the association between imputed SNPs and VT risk, after adjusting for principal components.

(d) Imputation quality criterion (r^2^).

(e) Combined meta-analysis p-value obtained using the Mantel-Haenszel inverse-variance weighting method.

**Table 2:** Association of haplotypes derived from rs2856650, rs3740689 and rs10769258 with VT risk in two GWAS.

Polymorphisms			GWAS from [2]			GWAS from [1]		
			Haplotype Frequencies		OR [95%CI]	Haplotype Frequencies		OR [95%CI]
rs2856650	rs3740689	rs10769258	Controls (n = 1002)	Cases (n = 1542)		Controls (n = 1218)	Cases (n = 411)	
G	G	T	0.356	0.329	reference	0.332	0.335	reference
G	A	T	0.282	0.283	1.07 [0.93–1.24] p = 0.316	0.298	0.301	0.99 [0.82–1.22] p = 0.983
G	A	C	0.049	0.041	0.91 [0.68–1.21] p = 0.521	0.046	0.037	0.79 [0.51–1.23] p = 0.292
A	G	T	0.021	0.058	2.99 [2.02–4.44] p = 4.23 10^−8^	0.017	0.040	2.37 [1.36–4.15] p = 2.39 10^−3^
A	A	C	0.260	0.260	1.07 [0.93–1.24] p = 0.341	0.275	0.261	0.94 [0.76–1.15] p = 0.545
A	A	T	0.019	0.017	0.99 [0.59–1.66] p = 0.964	0.019	0.013	0.65 [0.27–1.57] p = 0.335
Global test of haplotypic association			χ^ 2^ = 40.38 with 5 df p = 1.25 10^−7^			χ^2^ = 11.24 with 5 df p = 0.046		

The meta-analysis of the AGT haplotype-associated ORs obtained in the two GWAS samples lead to an overall OR of 2.78 [2.01–3.81] (p = 4.72 10^−10^). In a combined analysis of the individual-level genotype data of the two GWAS, the AGT haplotype frequency was estimated to 0.054 and 0.019 in cases and controls, respectively. This led to a combined OR of 3.03 [2.23–4.10] (p = 8.96 10^−13^) compared to the most frequent GGT haplotype (with estimated frequency 0.330 and 0.343 in cases and controls, respectively).

To replicate the association observed at the 11p11.2 locus, we genotyped two SNPs selected from Table 1, the *MYBPC3* rs2856656 and the *CELF1* rs60206633, in an independent sample of 592 controls and 596 VT cases from the FARIVE study [1]. We opted for the genotyping of two SNPs to further validate our hypothesis that the detected imputed rare variants were in strong LD. In FARIVE, the two SNPs were indeed in strong LD (D' = +0.89, r^2^ = 0.49) and showed evidence for association with VT (Table 3). In particular, the rs2856656-G allele was associated with an OR of 1.70 [1.15–2.51] (P = 0.008; P_Bonferroni-corrected_  = 0.016). This pattern of association was very consistent with that observed with the imputed SNPs of the long range LD block (Tables 1 & 2). Haplotype analysis of these two SNPs further showed that the rs2856656-G allele was carried by two haplotypes that were both more frequent in cases than in controls (Table S2). One of these two haplotypes also carried the rare rs60206633-G allele suggesting that the association signal observed at rs60206633 was due to its LD with rs2856656.

**Table 3:** Association of *MYPBC3* rs2856656 and *CELF1* rs60206633 with VT in the FARIVE study.

rs2856656	Controls	Cases	rs60206633	Controls	Cases
AA	510 (93%)	487 (88%)	CC	529 (94%)	527 (92%)
AG	38 (7%)	66 (12%)	CG	30 (6%)	46 (8%)
GG	2 (<1%)	2 (<1%)	GG	1 (<1%)	2 (<1%)
MAF(a)	0.038	0.063		0.029	0.044
Cochran-Armitage Trend test	p = 0.008			p = 0.062	

(a) Minor Allele Frequencies.

To further study the association of the 11p11.2 locus with VT, we genotyped the *MYBPC3* rs2856656 and the *CELF1* rs60206633 in the MARTHA study [1] composed of 1150 VT cases, among which 812 were part of the second GWAS, and a new sample of 801 controls that were not part of any of the two GWAS. By design, the control group of the MARTHA study had been enriched in healthy individuals heterozygous for the FV Leiden or FII G20210A mutations (∼40% of all controls). The association of rs2856656 with VT risk was not significant in MARTHA. While the frequency of the rs2856656-G allele in cases was comparable to that of other VT samples, in contrast it was higher than expected in controls (Minor Allele Frequency  = 0.069). Because of the enrichment of MARTHA controls in FV and FII carriers, we further investigated the genotype distribution of the rs2856656 according to the presence of FV and FII mutations and observed that the frequency of the rs2856656-G allele was strongly increased in FII carriers in all subgroups (Table 4). Conditioning on FII G20210A mutation, the allele frequency of the rs2856656 did not differ according to VT status (Table 4). In FARIVE, a conditional logistic regression analysis was conducted and revealed that, after adjusting for the FII G20210A (rs1799963), the rs2856656-G allele was no longer associated with VT risk (OR  = 1.26 [0.80–1.99], p = 0.310). The rs1799963 variant is located on chromosome 11 at a distance of 612 kb from rs2856656. The pairwise LD r^2^ between these two SNPs were 0.53 and 0.24 in MARTHA and FARIVE, respectively, while the associated D' were +0.86 and +0.68, respectively.

**Table 4:** Genotype distribution of the *MYPBC3* rs2856656 according to VT status and carrier-ship of F5/F2 mutations in the MARTHA and FARIVE studies.

	MARTHA Controls			MARTHA VT Cases		
rs2856656	Non carriers	FV Leiden carriers	FII G20210A carriers	Non carriers	FV Leiden carrier	FII G20210A carriers
AA	458 (97%)	174 (99%)	49 (35%)	563 (99%)	377 (98%)	53 (33%)
AG	13 (3%)	2 (1%)	91 (65%)	6 (1%)	8 (2%)	106 (67%)
GG	1 (<1%)	0 (-)	1 (<1%)	1 (<1%)	0 (-)	0 (-)
MAF(a)	0.016	0.006	0.333	0.007	0.010	0.333

	FARIVE Controls			FARIVE VT Cases		
rs2856656	Non carriers	FV Leiden carriers	FII G20210A carriers	Non carriers	FV Leiden carriers	FII G20210A carriers
AA	478 (94%)	24 (100%)	5 (33%)	427 (93%)	48 (91%)	8 (24%)
AG	28 (6%)	0 (-)	9 (60%)	32 (7%)	5 (9%)	24 (73%)
GG	1 (<1%)	0 (-)	1 (7%)	0 (-)	0	1 (3%)
MAF	0.030	0.00	0.367	0.035	0.047	0.394

(a) Minor Allele Frequencies.

**Figure 3:**
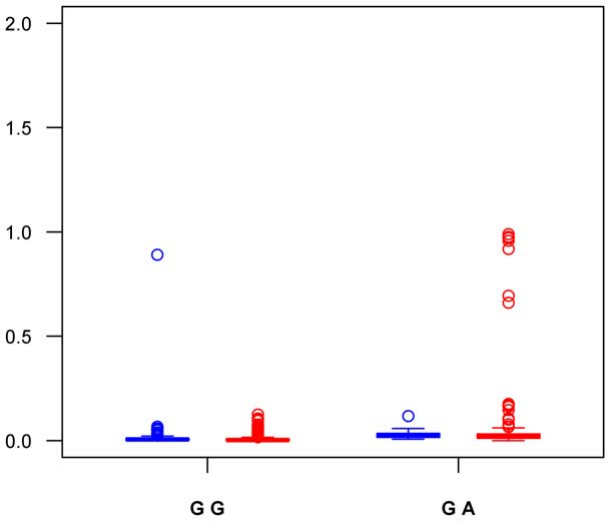
Box-Plot representation of the imputed dose at rs1799963 (FII G20210A) according to measured genotypes in a sample of 1,961 VT cases. The imputed dose in the 419 VT cases of the first GWAS is shown in blue while results obtained in the 1,542 VT part of the second GWAS are shown in red.

**Figure 4:**
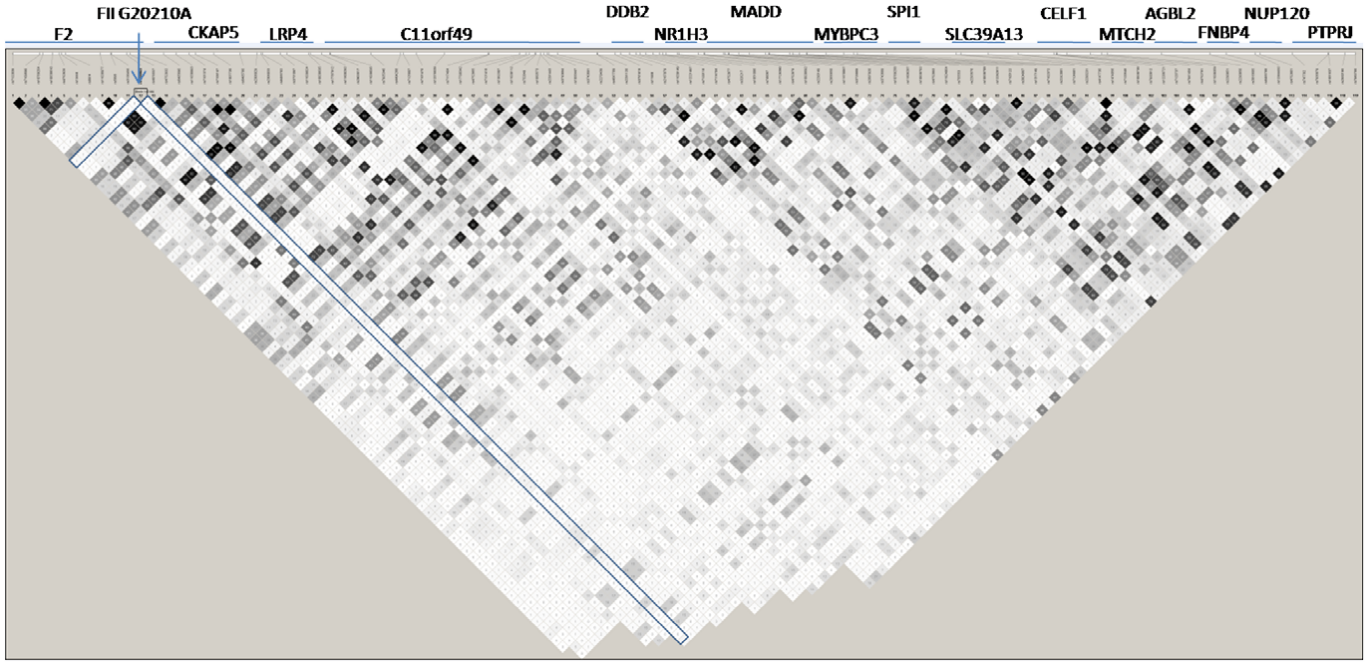
Pairwise linkage disequilibrium r^2^ between genotyped SNPs mapping from *F2* to *PTPRJ* genes at the 11p11.2 locus in a sample of 1,542 VT cases.

Coming back to our imputation GWAS data, we observed that the rs1799963 variant was imputed with poor quality (r^2^ = 0.12 and r^2^ = 0.27 in the first and second GWAS, respectively) and thus did not pass the quality control. Nevertheless, it showed suggestive association with VT, P = 0.053 and P = 0.110, in the first and second GWAS, respectively, for a combined statistical evidence of P = 0.020. A conditional logistic regression analysis was then conducted to estimate the effect of the imputed rs2856656 after adjusting for the imputed rs1799963. As indicated in Table 5, the imputed rs2856656-G allele was still associated with increased risk for VT, P = 7.22 10^−4^ and P = 1.13 10^−11^, in the first and second GWAS, respectively. However, because the rs1799963 variant was typed in the GWAS patients as part of the inclusion/exclusion criteria (see Materials and Methods), we re-ran the conditional analyses using the true genotyped rs1799963 in cases rather than the imputed dose. The association of rs2856656 with VT was now no longer significant, P = 0.643 and P = 0.122, in the first and second GWAS respectively (Table 5). To corroborate the poor quality of the imputation at rs1799963 mentioned above, we calculated the Spearman correlation between the imputed dose and the true genotype at rs1799963 in all cases for whom both information was available (Figure 1). This correlation was only ρ = 0.36 and ρ = 0.48 in the 419 and 1,542 cases from the first and second GWAS, respectively. As shown in Figure 3, the rs1799963 genotype was poorly imputed in heterozygotes individuals. Finally, a further haplotype analysis revealed that the rare rs17999963-A allele was mainly carried by the AGT at-risk haplotype discussed above (Table S3). Of note, a LD analysis of the whole chromosome 11 region from 46,600,000 to 48,000,000 bp containing 119 SNPs (Figure 4) showed that the rs1799963 variant was not in strong pairwise LD with any other common SNPs, the higher observed r^2^ being 0.15 with *AGBL2* rs7930612.

To summarize, in the two discovery GWAS based on imputed data as well as in the two standard case-control samples where the rs2856656 and the rs1799963 were directly genotyped, the association of rs2856656 with VT vanished after adjusting for rs1799963.

Finally, we re-analyzed the two imputed GWAS datasets by conditioning on the previously identified VT-associated SNPs at *ABO, HIVEP1, FV, F11, FGG, PROCR* and *STAB2* loci [2]. Except the 11p11.2 locus that remained genome-wide significant, no other locus demonstrated statistical association at P<1.0 10^−6^.

**Table 5:** Association of the imputed rs2856656 with VT risk in two GWAS datasets.

Allelic Odds Ratio [95% Confidence Interval	GWAS from ^(1)^ 419 cases/1,228 controls	GWAS from ^(2)^ 1,542 cases/1,110 controls
Crude	3.462 [1.754–6.835] p = 4.82 10^−4^	4.943 [2.987–8.180] p = 2.76 10^−12^
Adjusted		
- for rs1799963 imputed in controls and cases	3.211 [1.632–6.313] p = 7.22 10^−4^	5.469 [3.348–8.932] p = 1.13 10^−11^
- for rs1799963 imputed in controls and genotyped in cases	1.264 [0.469–3.402] p = 0.643	1.732 [0.864–3.474] p = 0.122

## Discussion

**Figure 5:**
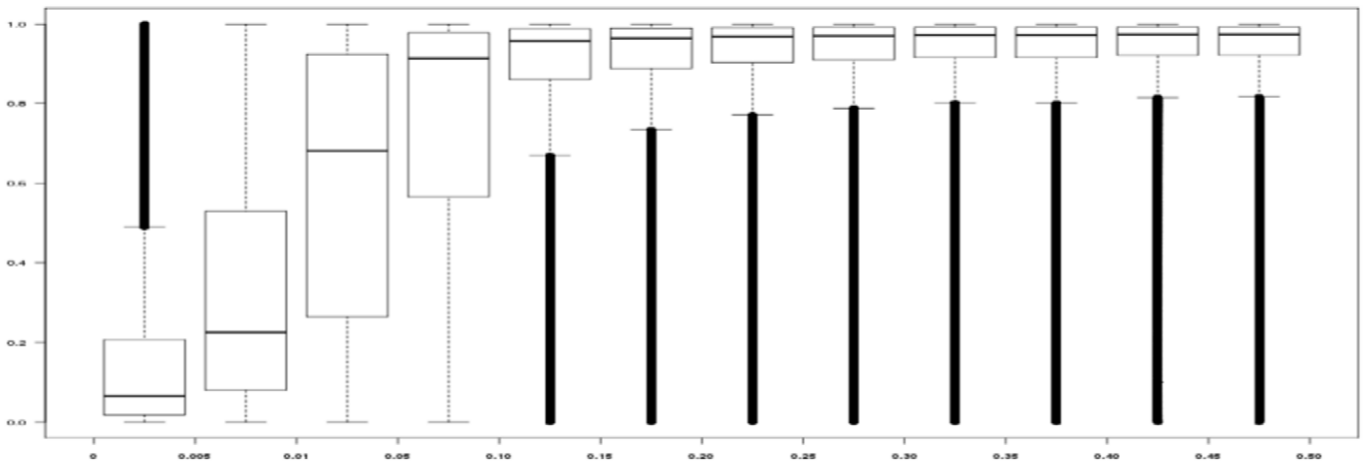
Box-Plot representation of the imputation quality (r^2^) according to the minor allele frequency of the SNPs inferred from 1000G 2010-08 release. Box-plot derived from the imputation analysis of the largest GWAS ^(2)^.

By conducting an updated and comprehensive in-depth analysis of two GWAS, we were able to “re-discover” a strong risk locus for VT known for more than one decade [15], the *F2* gene, but missed by all large scale association studies conducted so far on the disease [1], [2], [16]. Several conclusions can be drawn from this work. First, it adds to the rather limited illustrative literature about the interest of imputation-based GWAS analyses using the 1000 Genomes project that can help identify rare variants in new disease-associated loci not detected by the first waves of GWAS; Second, the functional variant could be quite far away from the detected hits. In our example, the original association signal mapped to an interval from 47,373,425 bp (*MYBPC3*) to 48,064,194 bp (*PTPRJ*) on chromosome 11, and this is up to 1.3 Mb away from the functional G20210A mutation. Would *PTPRJ* have been a plausible biological candidate for VT, our quest for the culprit variant could have led us to a dead end; Third, a functional variant could be missed if its imputation quality is low which would likely be the case for a non genotyped rare variant showing low to modest pairwise LD with other SNPs in its neighborhood. As shown in Figure 5, imputation quality was satisfactory for SNPs with inferred MAF >0.01. About 75% of the SNPs with MAF <0.01 demonstrated poor imputation quality in our study while ∼82% of the SNPs with MAF >0.01 were correctly (r^2^>0.3) imputed. Rare variants, like FII G20210A, which are not present on genotyping arrays can nevertheless be tagged by haplotypes generated from common SNPs not necessarily in strong LD with each other. A similar phenomenon was previously observed at the *LPA* locus associated with coronary artery disease [13]. From a population genetics perspective, it would be interesting to investigate whether evolutionary selection forces could be exerted on the *F2* locus as suspected for the *F5* gene [17], [18] and could explain why a functional “deleterious” allele was maintained on long-range haplotype.

To conclude, we have shown how a comprehensive analysis of 1000G imputed genotype data was able to discover a disease locus missed by previous GWASes. Our work also demonstrates the need for exercising careful analysis of detected imputed rare associations to avoid false inference on the functional variant, and for this, LD and haplotype analyses of the associated loci may be of great value. This may have both scientific and methodological utility for geneticists involved in similar studies.

## Materials and Methods

### Ethics Statement

Each individual study was approved by its institutional ethics committee and informed written consent was obtained in accordance with the Declaration of Helsinki. All subjects were of European origin. All subjects were of European origin.

Ethics approval were obtained :

for MARTHA, from the “Departement santé de la direction générale de la recherche et de l'innovation du ministère” (Projects DC: 2008-880 & 09.576).
for FARIVE, from the “Comité consultatif de protection des personnes dans la recherche biomedicale” (Project n° 2002-034).
for the 3C study, from the institutional ethics committees of the Kremlin-Bicetre Hospital.

### Studied populations

The description of the studied populations has already been extensively described in [1] for the first GWAS MARTHA and FARIVE studies, and in [2] for the second GWAS.

Briefly, all studied patients were unrelated Caucasians subjects with a document first event of VT and lacking strong known genetic risk factors, including AT, PC, or PS deficiencies, and homozygosity for FV Leiden or FII 20210A mutation. Cases (n = 453) from the first GWAS were patients with early age of onset of VT (<50 years) recruited in 4 different French centers (Grenoble, Marseille, Montpellier, and Paris), and were compared to 1,327 national French controls [3] with no chronic conditions, and no regular medicines. Patients of the second GWAS (n = 1,597) were recruited from the Thrombophilia center of La Timone hospital (Marseille, France) with no restriction one age of onset, and were compared to another independent sample of 1,140 French controls free of any chronic disease and selected from the 3C study [4]. The MARTHA study was composed of 1,150 VT patients also recruited from the Thrombophilia center of La Timone hospital, among which 812 were part of the second GWAS, and of a control sample of 801 healthy individuals. 475 of these controls were recruited from the Marseille area and 326 were healthy heterozygous carriers of the FV Leiden or FII G20210A mutations selected from the national health examination centers of the French Social Security in collaboration with the Hemostasis and Thrombosis Study Group. The FARIVE study is a multicenter case-control study composed of 607 patients with a documented first episode of VT and 607 controls matched for age and sex.

### Genotyping and Quality control

#### First Genome Wide Association Study [1]

Individuals participating in this GWAS were genotyped for 317,139 SNPs using the Illumina Sentrix HumanHap300 Beadchip. The application of several quality control criteria previously described [1] lead the final analysis of 291,872 genotyped SNPs in a sample of 419 cases and 1,228 controls.

#### Second Genome Wide Association Study [2]

As extensively described in [2], 1011 VT patients were typed with the Illumina Human 610-Quad Beadchip and 586 VT patients were typed with the Illumina Human 660W-Quad Beadchip. Healthy individuals from the 3C study were typed with the Illumina Human 610-Quad Beadchip. The application of quality control filters lead to the final analysis of 481,0002 autosomal SNPs in a sample of 1,542 VT patients and 1,110 healthy individuals.

#### Replication studies

In FARIVE and MARTHA, the *MYBPC3* rs2856656 and the *CELF1* rs60206633 were genotyped by allele-specific PCR.

### Statistical Analysis

#### Imputation

In both GWAS datasets, imputation of 11,572,501 autosomal SNPs was conducted using the MACH [11] according the 1000G 2010-08 release reference dataset. The association of each imputed SNP with VT was tested by use of a logistic regression analysis in which allele dosage (from 0 to 2 copies of the minor allele) of imputed SNPs was used. Analyses were adjusted for the first four principal components and were performed using the mach2dat (v 1.0.18) software (http://www.sph.umich.edu/csg/abecasis/MACH/download/).

#### Combined GWAS analysis

All SNPs with acceptable imputation quality (r^2^>0.3) in both imputed GWAS datasets were entered into a meta-analysis, leading to 6,754,935 SNPs left for statistical association analysis. For the meta-analysis, a fixed-effect model relying on the inverse-variance weighting was used as implemented in the METAL software [12]. Homogeneity of associations across the two GWAS studies was tested using the Mantel-Haenszel method [19]. A statistical threshold of 7.4 10^−9^, which controls for the Bonferroni corrected type I error rate of 0.05 according to the number of tested SNPs, was used to declare genome-wide significance.

#### Haplotype analysis

A systematic analysis of all possible combinations of 1 to 4 typed SNPs within the chromosome 11 47,300,000–48,100,000 interval was performed to select the most informative and parsimonious haplotype configuration in terms of predicting disease status using a previously described strategy based on the Akaike's Information Criterion (AIC) [13]. This strategy calculates an AIC value for each investigated haplotype model and then subtracts from each model the minimum AIC value obtained over all models explored, giving a rescaled AIC value for each haplotype model. The models with a rescaled AIC ≤2 are considered equivalent to the most informative model. Among these equivalent models, the most parsimonious model with the fewest polymorphisms is considered the best model. The THESIAS program implementing the stochastic-EM algorithm [14] for haplotype inference was used to estimate haplotype frequencies and haplotype effects on VT risk derived from specific sets of SNPs.

#### Replication

In the FARIVE and MARTHA studies, association of tested SNPs with VT risk was assessed by use of the Cochran-Armitage trend test [20] and logistic regression models.

## Supporting Information

Figure S1 **Distribution of the inferred minor allele frequencies in two imputed GWAS datasets.** Only SNPs with imputation quality control r^2^ > 0.30 are represented.
(TIF)

Figure S2 **Quantile-Quantile plots summarizing the results of a meta-analysis of two GWAS for VT.** QQ plot derived from SNPs imputed according to 1000G 2010-08 release is shown in blue with its 95% confidence interval in shaded area. The corresponding genomic control coefficient was 0.993. QQ plot derived from HapMap2 release 21 imputed data is shown in black with a genomic control of 1.023.
(TIF)

Table S1 **Genome-wide significant (p<7.4×10–9) SNPs associated with VT risk in a meta-analysis of two imputed GWAS datasets.** Results are shown in a separate EXCEL file (Table S1). Beta: log Odds Ratio for VT risk associated with the A2 allele SE: Standard error of the beta coefficient r^2^: imputation quality control coefficient.
(XLSX)

Table S2 **Haplotype frequencies distribution derived from the** ***MYBPC3*** **rs2856656 and** ***CELF1*** **rs60206633 according to VT status in the FARIVE study.**
(DOCX)

Table S3 **Main haplotype frequencies distribution derived from the rs2856650, rs3740689, rs10769258, and rs1799963 in two samples of genotyped VT patients.**
(DOCX)
