# Supplementary figures and images for: Caution in Interpreting Results from Imputation Analysis When Linkage Disequilibrium Extends over a Large Distance: A Case Study on Venous Thrombosis
Source: PLoS One. 2012 Jun 4;7(6):e38538. doi: 10.1371/journal.pone.0038538 (PMC3366937; doi:10.1371/journal.pone.0038538)

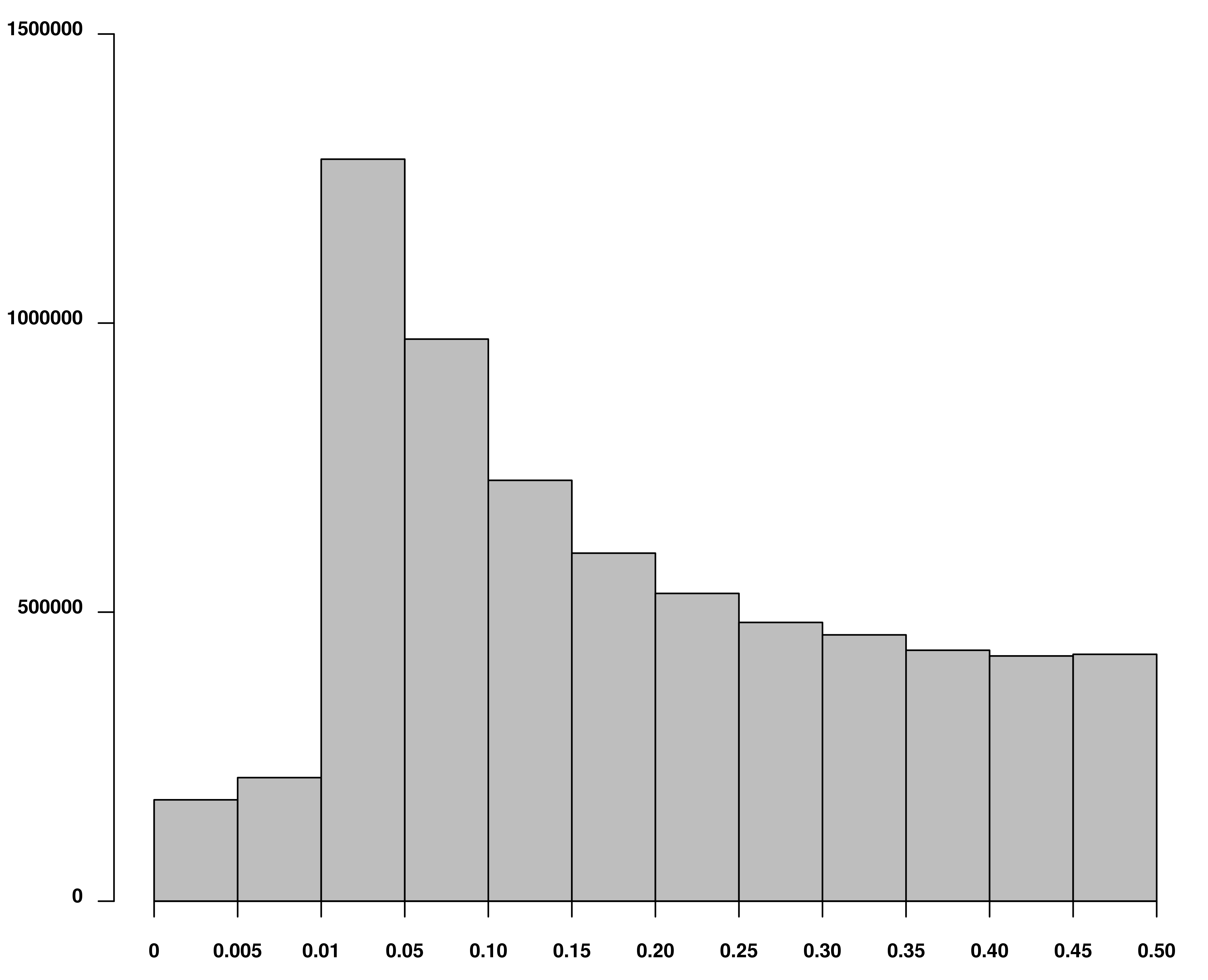

Supplement: Figure S1 — Distribution of the inferred minor allele frequencies in two imputed GWAS datasets. Only SNPs with imputation quality control r2 > 0.30 are represented. (TIF) [file pone.0038538.s001.tif]

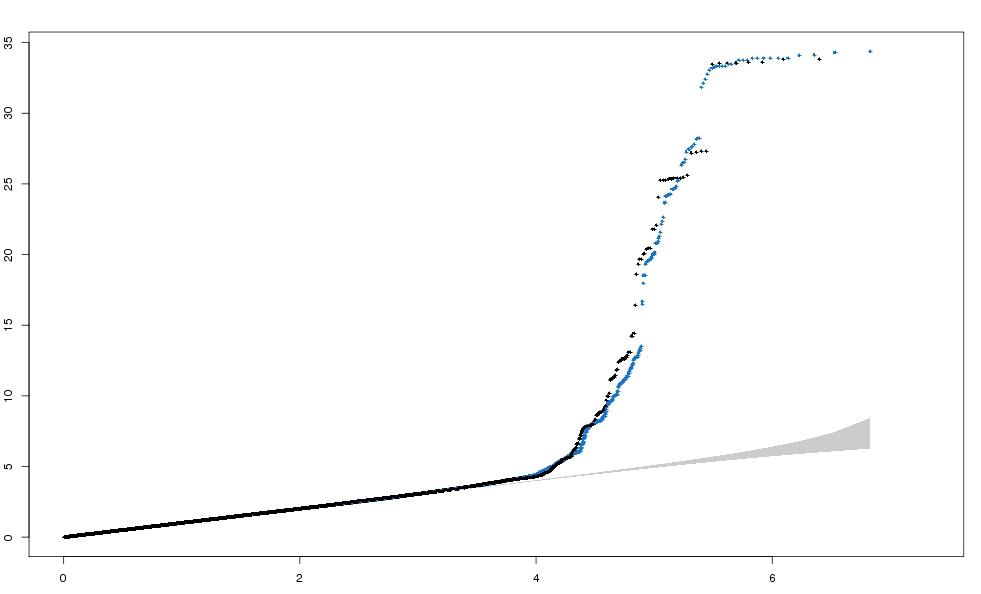

Supplement: Figure S2 — Quantile-Quantile plots summarizing the results of a meta-analysis of two GWAS for VT. QQ plot derived from SNPs imputed according to 1000G 2010-08 release is shown in blue with its 95% confidence interval in shaded area. The corresponding genomic control coefficient was 0.993. QQ plot derived from HapMap2 release 21 imputed data is shown in black with a genomic control of 1.023. (TIF) [file pone.0038538.s002.tif]
