# Supplementary material for: Caution in Interpreting Results from Imputation Analysis When Linkage Disequilibrium Extends over a Large Distance: A Case Study on Venous Thrombosis
Source: PLoS One. 2012 Jun 4;7(6):e38538. doi: 10.1371/journal.pone.0038538 (PMC3366937; doi:10.1371/journal.pone.0038538)
Supplement: Table S2 — Haplotype frequencies distribution derived from the MYBPC3 rs2856656 and CELF1 rs60206633 according to VT status in the FARIVE study. (DOCX) [file pone.0038538.s004.docx]

**Table S2. Haplotype frequencies distribution derived from the *MYBPC3* rs2856656 and *CELF1* rs60206633 according to VT status in the FARIVE study**

| Polymorphisms | | Haplotype Frequencies | |
| --- | --- | --- | --- |
| rs2856656 | rs60206633 | Controls n = 548 | VT Cases n = 555 |
| A | C | 0.950 | 0.931 |
| G | C | 0.016 | 0.026 |
| G | G | 0.034 | 0.043 |
