# Supplementary material for: Caution in Interpreting Results from Imputation Analysis When Linkage Disequilibrium Extends over a Large Distance: A Case Study on Venous Thrombosis
Source: PLoS One. 2012 Jun 4;7(6):e38538. doi: 10.1371/journal.pone.0038538 (PMC3366937; doi:10.1371/journal.pone.0038538)
Supplement: Table S3 — Main haplotype frequencies distribution derived from the rs2856650, rs3740689, rs10769258, and rs1799963 in two samples of genotyped VT patients. (DOCX) [file pone.0038538.s005.docx]

**Table S3. Main haplotype frequencies distribution derived from the rs2856650, rs3740689, rs10769258, and rs1799963 in two samples of genotyped VT patients.**

| rs2856650 | rs3740689 | rs10769258 | rs1799963 (FII G20210A) | Haplotype frequencies | |
| --- | --- | --- | --- | --- | --- |
|  |  |  |  | N = 411 patients  from GWAS ^(1)^ | N = 1,542 patients  from GWAS ^(2)^ |
| G | G | T | G | 0.333 | 0.322 |
| G | A | T | G | 0.298 | 0.277 |
| G | A | C | G | 0.037 | 0.042 |
| A | G | T | **A** | 0.037 | 0.046 |
| A | G | T | G | 0.015 | 0.010 |
| A | A | C | **A** | 0.005 | 0.010 |
| A | A | C | G | 0.257 | 0.248 |
| A | A | T | G | 0.014 | 0.018 |

(1). Tregouet DA et al. (2009) Blood 113: 5298-5303.

(2) Germain M et al. (2011) Plos One 6: e25581.
